# Plasticity and resistance of cancer stem cells as a challenge for innovative anticancer therapies – do we know enough to overcome this?

**DOI:** 10.17179/excli2024-6972

**Published:** 2024-02-29

**Authors:** Agnieszka Knopik-Skrocka, Alicja Sempowicz, Oliwia Piwocka

**Affiliations:** 1 Department of Cell Biology, Faculty of Biology, Adam Mickiewicz University of Poznań, Poland; 2 Section of Regenerative Medicine and Cancer Research, Natural Sciences Club, Faculty of Biology, Adam Mickiewicz University, 61-614 Poznań, Poland; 3 Radiobiology Laboratory, Department of Medical Physics, Greater Poland Cancer Center, Poznań, Poland; 4 Department of Electroradiology, Poznan University of Medical Sciences, Poznań, Poland; 5 Doctoral School, Poznan University of Medical Sciences, Poznań, Poland

**Keywords:** cancer stem cells, tumor heterogeneity, hypoxia, plasticity, immunosuppression, resistance, therapies

## Abstract

According to the CSC hypothesis, cancer stem cells are pivotal in initiating, developing, and causing cancer recurrence. Since the identification of CSCs in leukemia, breast cancer, glioblastoma, and colorectal cancer in the 1990s, researchers have actively investigated the origin and biology of CSCs. However, the CSC hypothesis and the role of these cells in tumor development model is still in debate. These cells exhibit distinct surface markers, are capable of self-renewal, demonstrate unrestricted proliferation, and display metabolic adaptation. CSC phenotypic plasticity and the capacity to EMT is strictly connected to the stemness state. CSCs show high resistance to chemotherapy, radiotherapy, and immunotherapy. The plasticity of CSCs is significantly influenced by tumor microenvironment factors, such as hypoxia. Targeting the genetic and epigenetic changes of cancer cells, together with interactions with the tumor microenvironment, presents promising avenues for therapeutic strategies.

See also the Graphical abstract(Fig. 1).

## Introduction

**Figure 2:**
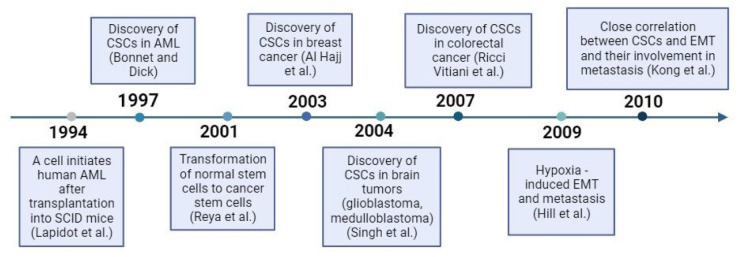
The history of CSCs discovery and their role in metastasis (Capp, 2019; Lachat et al., 2021, changed).

The concept of cancer stem cells (CSCs) emerged from investigations into the origins of malignant tumor formation. Landmark studies on acute myeloid leukemia provided evidence of CSCs and their fundamental role in tumor initiation and progression (Lapidot et al., 1994[70]). Subsequently, CSCs were identified in solid tumors such as breast cancer (Al-Hajj et al., 2003[3]), glioblastoma (Singh et al., 2004[121]), and later, colon cancer (Ricci-Vitiani et al., 2007[111]). The identification of CSCs relies on biological and molecular characteristics associated with a stem cell phenotype, including the expression of specific clusters of differentiation (CD) markers, such as CD133. Remarkably, studies demonstrated that as few as 100 cells expressing CD133 were sufficient to initiate brain tumor growth, while cells lacking CD133 expression, even in quantities ranging from 50,000 to 100,000, failed to induce tumor growth (Singh et al., 2004[121]). Consistent findings were observed in studies involving xenografts of human bladder, colorectal, and pancreatic tumors (Baccelli and Trumpp, 2012[10]). In leukemia, the number of CSCs ranges from approximately 0.1 % to 1 %, while in breast cancer it is around 2 %. Similarly, colorectal cancer exhibits CSC proportions of 0.5 % to 1 %. In particular, in triple negative breast cancer, characterized by its high aggressiveness, the proportion of CSCs can exceed 10 % of tumor cells (Jagguppilli and Elkord, 2012[55]). Figure 2(Fig. 2) (References in Figure 2: Capp, 2019[20]; Lachat et al., 2021[67]) shows a timeline that highlights key milestones in the history of CSC research and its involvement in metastasis.

The discoveries mentioned above laid the foundation for the CSC hypothesis, which forms the core of the hierarchical model of cancer (Rich, 2016[112]). CSCs have become a focal point of intensive biomedical research aimed at comprehensively understanding their characteristics. These cells share numerous traits with normal stem cells, including the expression of various surface markers (Atashzar et al., 2020[8]; Walcher et al., 2020[139]). Despite the increasing knowledge about CSCs, their origin remains elusive. Recently, attention has shifted to the potential involvement of the dedifferentiation process, wherein mutant progenitor cells or mutant differentiated somatic cells transform into a cancer stem-like state (Senga and Grose, 2021[115]; Walcher et al., 2020[139]).

Like normal stem cells, CSCs possess the ability for self-renewal and unlimited proliferation (Batlle and Clever, 2017[12]; Walter et al., 2021[140]). However, CSCs exhibit genetic instability, such as mutations, and undergo numerous epigenetic changes, including the down- or upregulation of non-coding RNAs (ncRNAs) (Bryl et al., 2022[17]; Khan et al., 2019[62]). These alterations contribute to the high plasticity of CSCs, both metabolically and phenotypically, enabling them to undergo epithelial-mesenchymal transition (EMT) (Gupta et al., 2019[46]; Lachat et al., 2021[67]; Thankamony et al., 2020[131]). The strong association between CSC stemness and the ability to undergo EMT is corroborated by the expression of genes such as Snai1, Twist, Zeb1/2, and the activation of signaling pathways like Notch, Hedgehog, MAPK, PI3K/AKT, Wnt, as well as the HIF-1 (hypoxia-inducible factor 1) pathway (Lachat et al., 2021[67]).

Many of the hallmarks of cancer (Hanahan and Weinberg, 2000[50], 2011[49]; Hanahan, 2022[48]) can be attributed to CSCs. These cells can stimulate VEGF-dependent neoangiogenesis and actively participate in alternative tumor vascularization, known as vasculogenic mimicry (VM) (Knopik-Skrocka et al., 2017[64]). CSCs interact with the microenvironment through various mechanisms and evade immune surveillance (Tsuchiya and Shiota, 2021[134]; Xie et al., 2022[154]; Yang and Teng, 2023[156]). Consequently, CSCs exhibit high resistance to chemotherapy, radiotherapy, and immunotherapy. Therefore, innovative anticancer therapies should target CSCs.

## The Origin of CSCs and their Markers

**Figure 3:**
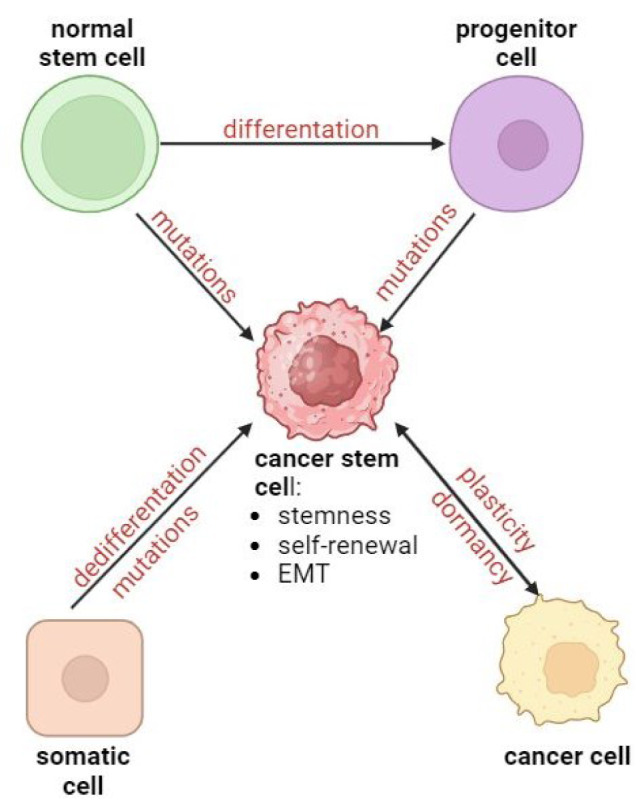
Hypothesis of CSC origin (Khan et al., 2019; Walcher et al., 2020, changed).

Research on the origin of CSCs is strongly related to the elucidation of cancer initiation and progression. A stochastic model has prevailed for many years, assuming every cancer cell has tumorigenic potential. The discovery of CSCs resulted in a change in this model, and the so-called hierarchical model was proposed, stating that only CSCs can initiate tumor growth at both the primary and metastatic sites (Fanali et al., 2014[39]). To date, it has not been possible to determine the origin of CSCs. It is assumed that they may come from normal stem cells, progenitor cells, or differentiated somatic cells undergoing mutations, as well as from normal cancer cells (Figure 3(Fig. 3); References in Figure 3: Khan et al., 2019[62]; Walcher et al., 2020[139]). Regarding the high capacity of cancer cells to change their state, a dormancy process can be important as a protective mechanism against stress (Talukdar et al., 2019[127]). 

With their long lifespan, stem cells are susceptible to accumulating mutations, potentially leading to cancerous transformation. CSCs are characterized by activating genes such as c-Myc, KLF-4, Sox-2, and OCT-4 (Villodre et al., 2019[137]), which may contribute to oncogenesis under favorable microenvironmental conditions. Notably, OCT-4 serves as a biomarker in seminomas, Sox-2 acts as a "key driver" in breast cancer and brain tumors, while KLF-4 is associated with colorectal cancer (Senga and Grose, 2021[115]).

The origin of CSCs from stem cells may be supported by their shared expression of surface markers typical of normal stem cells (Atashzar et al., 2020[8]). After undergoing prior mutation, progenitor cells can be reprogrammed into CSCs (see Figure 3(Fig. 3)). Notably, normal cancer cells can reversibly transition into a parent state, indicating that CSCs can arise from non-CSCs (Lambert and Weinberg, 2021[69]). Mutations and pro-inflammatory factors can facilitate this transition between parental and non-parental phenotypes, with the acquisition of the cell's ability to undergo EMT being a crucial determinant in this process (Khan et al., 2019[62]).

Moreover, differentiated somatic cells could serve as another source of CSCs (Khan et al., 2019[62]). The role of dedifferentiation in CSC formation is evidenced by studies conducted on glioblastoma, colorectal cancer, and pancreatic cancer. For instance, the dedifferentiation of normal intestinal epithelial cells may lead to the generation of tumor-initiating stem-like cells (Shih et al., 2001[119]). According to this hypothesis, CSCs in colorectal cancer may arise from a "top-down" process involving the dedifferentiation of a mature intestinal epithelial cell from the top of the crypt. Similarly, the ability of differentiated epithelial cells to dedifferentiate towards intraepithelial neoplasia has been observed in pancreatic follicular cells (Senga and Grose, 2021[115]).

As previously mentioned, CSCs represent a small fraction of the tumor cell population and exhibit some similarities to normal stem cells, posing challenges in their isolation and identification. Presently, CSCs are isolated by exploiting the affinity of antibodies against CSC surface markers or based on physical parameters, such as density or size (Babaei et al., 2021[9]). Methods like FACS (Fluorescence-Activated Cell Sorting) and MACS (Magnetic-Activated Cell Sorting), which utilize surface antigens, have proven highly effective. In fact, the initial isolation of CSCs was accomplished through FACS using antibodies and fluorescent markers (Lapidot et al., 1994[70]). Table 1(Tab. 1) (References in Table 1: Atashzar et al., 2020[8]; Kim et al., 2020[63]; Walcher et al., 2020[139]) illustrates the primary surface markers of CSCs.

One of the most known surface markers of CSCs is CD133, which is also present in normal hemopoietic and neural stem cells (Glumac and LeBeau, 2018[42]). CD133 is a poor prognostic factor (Park et al., 2019[97]); its overexpression correlates positively with the tumor stage. CD133 also mediates the drug resistance of CSCs. Studies in lung cancer showed that CSCs characterized by a CD133+ phenotype exhibited increased expression of some ATP-binding cassette transporters (ABC) and resistance to paclitaxel and platinum (Li et al., 2021[75]). Zhou et al. (2022[161]) showed that silencing CD133 expression in gastrointestinal adenocarcinoma cells increased the sensitivity of these cells to chemotherapeutics and inhibited CSC invasiveness.

CD44 is a receptor for many extracellular matrix components and a co-receptor for growth factors and cytokines (Li et al., 2021[75]). CD44 is profoundly involved in tumor initiation and progression. The ability of CD44 to receive signals from the microenvironment and transmit them to membrane-associated cytoskeletal proteins or the nucleus has been demonstrated, which affects gene expression and thus, cell behavior (Li et al., 2021[75]). CD44, like CD133, affects cell resistance. Two types of CD44+ and CD44- cells were identified in ovarian cancer studies. Only cells with CD44 expression resisted chemotherapy (Alvero et al., 2009[5]). Interestingly, CD44 can interact with hyaluronic acid, which leads to the activation of the Nanog and STAT-3 pathways and the expression of one of the ABC transporter, MDR1 (Price et al., 2018[105]). MDR1 is directly involved in cancer chemoresistance and is an essential marker of these cells due to high expression in CSCs (Muriithi et al., 2020[90]; Zhou et al., 2001[162]). Some ABC proteins can transport glutathione and thus, indirectly maintain redox homeostasis inside the cell. ABC transporters may thus protect CSCs from oxidative stress-related damage (Begicevic and Falasca, 2017[13]).

The results of the meta-analysis concluded that the presence of CD24 on the surface of CSCs is a factor associated with poor prognosis, metastasis, and shorter survival (Lee et al., 2009[73]; Yaghjyan et al., 2022[155]). Like CD44, CD24 with CD133 and CD166 are highly expressed in many types of cancer. In ovarian cancer, CD166 promotes invasion and metastasis and can inhibit apoptosis (Kim et al., 2020[63]). In lung cancer, high expression of CD44 and CD166 is correlated with high EpCAM expression, promoting metastasis (Walcher et al., 2020[139]).

## Static or Dynamic State? A Controversy around CSC Hypothesis

Three models of tumor development were proposed - stochastic, hierarchical, and clonal evolution models (Cabrera et al., 2015[18]; Fanali et al., 2014[39]; Takebe and Ivy, 2010[126]). According to the stochastic model, all tumor cells have the same tumor-initiating activity, and the tumor mass is homogenous. The last two models assume the existence of CSCs and tumor heterogeneity. In the hierarchical model, only a small number of tumor cells (CSCs) show a capacity for tumor development and generate progeny cells, which lose tumorigenic potential (Lee et al., 2011[72]; O'Connor et al., 2014[95]; Rich, 2016[112]). The clonal evolution model indicates that CSCs can acquire selective oncogenic changes and then dominate other types of CSCs through active proliferation in a given tumor niche. Cells of the dominant subclones show similar tumorigenic potential (Nowell, 1976[94]). In each separate niche, such clonal expansions can occur independently (Takebe and Ivy, 2010[126]).

The lack of standardized, optimal study methods, unclear definitions, and nomenclature (Jordan, 2009[58]; Lathia, 2013[71]) seems to be the main problem in CSC studies. Different terms are used to describe cancer cells capable of self-renewal, such as stem-like tumor cells, tumor-initiating cells, or cancer stem cells (Lathia, 2013[71]). CSC best reflects the nature of these cells, which exhibit the ability to self-renewal and the capacity to recapitulate the parental tumor with cellular heterogeneity after transplantation to immunodeficient mice (Clarke et al., 2006[28]; Lathia, 2013[71]). However, the term cancer stem cell can be confusing (Jordan, 2009[58]). In light of data discussed in previous sections, CSCs can arise from stem cells and differentiated cancer cells forming a mass of tumors (non-CSCs). Studies on breast cancer (Chaffer et al., 2011[23]; Chaffer et al., 2013[24]) have revealed the acquisition of self-renewal capacity by non-CSCs. The switch to a CSC-like state depends on ZEB1, a transcription factor associated with EMT (Chaffer et al., 2013[24]). Interestingly, this conversion was mainly observed in basal, rather than in luminal type of breast cancer. It can explain the higher aggressiveness of the basal-like subtype of triple-negative breast cancer, compared to estrogen-dependent breast cancer (Dai et al., 2017[31]).

The reprogramming of non-CSCs to stem-like cells was also observed in glioblastoma ( Suvà et al., 2014[125]). Four transcription factors (POU3F2, SOX2, SALL2, OLIG2) introduced into differentiated glioblastoma cells induced stemness phenotype. It suggests that this state can be transient and reversible in CSCs and non-CSCs (O'Connor et al., 2014[95]). Hence, an alternative plasticity model of reversible cellular plasticity of cancer cells was proposed. This model focuses on a dynamic, not a static, CSCs model. Cancer stemness is related to the state of the cell rather than its type, and bidirectional interconversions between CSCs and non-CSCs are possible (Donnenberg et al., 2013[36]; Gupta et al., 2009[44]; O'Connor et al., 2014[95]; Rich, 2016[112]). Tumor microenvironment factors, like hypoxia, can be the driving force that reverts non-CSCs to a functional CSC state (Lathia, 2013[71]; Qin et al., 2017[106]).

A small number of CSCs within the tumor, raised in the hierarchical model, was questionable. The number of CSCs can vary significantly in different tumor types (Baccelli and Trumpp, 2012[10]). In some cancers, like melanoma, the cells responsible for tumor initiation were quite numerous (Quintana et al., 2008[108]). The frequency of CSCs can be increased during tumor progression and differ between grade 1 and grade 3 breast tumors (Pece et al. 2010[99]). The functional test is considered a standard in cancer cell stemness studies, however, its relevance is still debated (Lathia, 2013[71]). With this method, it is possible to measure the ability of transplanted cells to develop tumors in mice, but not the frequency of CSCs in tumors in situ (O'Connor et al., 2014[95]). This means that more precise tests are necessary to establish the number of cancer cells with tumorigenic potential. Recently, MutaSeq and mitoClone were used in acute myeloid leukemia to distinguish CSCs from non-CSCs and normal stem cells (Velten et al., 2021[135]). These tests are based on the genomic and mitochondrial mutations, not on CSC surface markers. Surface markers do not appear to be specific in distinguishing tumorigenic from non-tumorigenic cells (Lathia, 2013[71]; Quintana et al., 2010[107]; Shackleton et al., 2009[116]). CD133 is not exclusive to CSCs and can also be expressed in non-CSC populations (Li, 2013[76]). The studies of Shmelkov et al. (2008[120]) showed that CD133 expression in the colon is not restricted to CSCs, and CD133 is expressed on differentiated colonic epithelium. The use of surface markers does not allow for the tracking of dynamic changes, such as EMT, which is associated with acquiring stemness phenotype (Shibue and Weinberg, 2017[118]). Studies using reporter systems Oct4GFP and CatulinGFP help to observe the changing states of invasive cancer cells (Gielata et al., 2022[41]). RNAseq analysis of these cells revealed high expression of genes critical to cellular movement, invasion, and VM.

The CSC hypothesis is still being rebuilt, and it may be that the clonal evolution and hierarchical models together may explain what happens during tumor development. Genetically and epigenetically distinct cancer stem cell subclones, can derive from the cell with the first oncogenic mutation. Next, the subclones can give rise to more differentiated non-CSCs. Under tumor microenvironment influence, dynamic changes between stem (tumorigenic) and non-stem cell (non-tumorigenic) states may follow due to high cell plasticity and adaptation capabilities.

## CSC Plasticity

CSCs are characterized by high metabolic and phenotypic plasticity (Gupta et al., 2019[46]; Thankamony et al., 2020[131]). The concept of CSC plasticity has become a paradigm, guiding efforts to enhance our understanding of initiation processes, tumor progression, and the transition of cells into a dormant state under challenging conditions. This transition often results in resistance to treatment (Gupta et al., 2019[46]; Pastushenko et al., 2018[98]; Talukdar et al., 2019[127]; Thankamony et al., 2020[131]). The induction factor and driving force of CSC plasticity is hypoxia (Lachat et al., 2021[67]). Oxygen deficiency in the tumor leads to activation of HIF-1α and HIF-2α (hypoxia-inducible factors) (Hajizadeh et al., 2019[47]). In the normoxic state, the HIF-1α subunit is inactivated, while under hypoxic conditions, HIF-1α forms a complex with HIF-1β, acting as a transcription factor (Zhang et al., 2021[159]). HIF-1α preferentially increases the activity of genes associated with glycolysis, whereas HIF-2α activates genes mainly related to the cell cycle and stemness (Hajizadeh et al., 2019[47]). Table 2(Tab. 2) (References in Table 2: Chatterjee and Sil, 2019[25]; Clark and Palle, 2016[28]; Dong et al., 2016[35]; Gupta et al., 2019[46]; Hajizadeh et al., 2019[47]; Jin et al., 2020[56]; Sharma et al., 2022[117]; Sun et al., 2020[124]; Wang et al., 2021[143]; Wicks and Semenza, 2022[150]; Zhang et al., 2021[159]) shows some of the effects of hypoxia induced gene expression.

### CSC metabolic plasticity 

Tumor cells can alter their metabolism toward glycolysis in hypoxia conditions (Warburg et al., 1927[147]). This phenomenon was termed aerobic glycolysis, or the Warburg effect. The metabolic plasticity of cancer cells has become one of the hallmarks of cancer (Hanahan and Weinberg 2000[50], 2011[49]) and is associated with the heterogeneity of cancer cells. High metabolic adaptability is demonstrated by CSCs (Luo and Wicha, 2015[83]; Peiris-Pagès et al., 2016[100]; Thankamony et al., 2020[131]). Simultaneously, researchers have highlighted that non-CSCs can develop stem-like characteristics by altering their metabolism, a phenomenon called “metabostemness” (De Francesco et al., 2018[32]).

According to Peires-Pagès et al. (2016[100]), the metabolism of cancer cells is determined by their degree of differentiation. For proliferating non-CSCs, glycolytic metabolism is typical. These cells show a high glucose demand and low oxygen consumption. Acquisition of large amounts of glucose is possible due to increased expression of the GLUT1 transporter (Jin et al., 2020[56]). CSCs that are in a quiescent state perform OXPHOS-based metabolism. In addition, these cells can cooperate with microenvironment cells, such as cancer-associated fibroblasts (CAFs), to obtain lactate (reverse Warburg effect) and convert it into pyruvate, which is used to trigger the Krebs cycle. The quiescent state is reversible and CSCs can then enter the path of proliferation, accompanied by a change in metabolism. CSCs in this state can benefit from aerobic glycolysis and OXPHOS, and rely on the reverse Warburg effect (Peires-Pagès et al., 2016[100]).

The metabolic states of cancer cells described above correspond to three recently proposed metabolic patterns (Warrier et al., 2023[148]), based on a mathematical model verifying HIF-1 and adenosine 5'monophosphate-activated protein kinase (AMPK) activity. AMPK acts as a regulator of mitochondrial respiration. The first pattern is glycolytic phenotype with high HIF-1 and low AMPK activities (proliferative non-CSCs), the second OXPHOS phenotype with high HIF-1 and low AMPK activities (quiescent CSCs), and the third mixed glycolysis/OXPHOS phenotype with high HIF-1 and high AMPK activities (proliferative CSCs). 

### Neoangiogenesis and VM 

The glycolytic metabolic pattern of cancer cells is associated with increasing hypoxia. Under these conditions, the gene encoding VEGF is activated by HIF-1. VEGF forms tumor blood vessels by sprouting angiogenesis, which enables tumor cell survival and metastasis (Knopik-Skrocka et al., 2017[64]; Nicolas et al., 2019[92]). Due to their abnormal structure, tumor vessels have slow blood flow and thus poor access to intravenously administered chemotherapeutic agents (Knopik-Skrocka et al., 2017[64]). An alternative VM vascularization may be induced in VEGF deficiency after antiangiogenic therapy with Bevacizumab. A close correlation has been shown between VM channel formation and HIF-1 activity (Angara et al., 2017[6]). Maniotis et al. (1999[86]) first described the VM process in uveal melanoma. CSCs expressing CD133, Sox-2, and Twist are directly involved in the formation of channels of vascular-like structures (Lai et al., 2012[68]; Wang et al., 2015[141]; Wei et al., 2021[149]). During the VM process, CSCs can transdifferentiate towards endothelial cells (ECs), expressing CD31 and CD105 on their surface (Soda et al., 2011[122]). The ability of CSCs to transdifferentiate indicates their high plasticity. 

### EMT as a “hallmark” of CSC phenotypic plasticity

**Figure 4:**
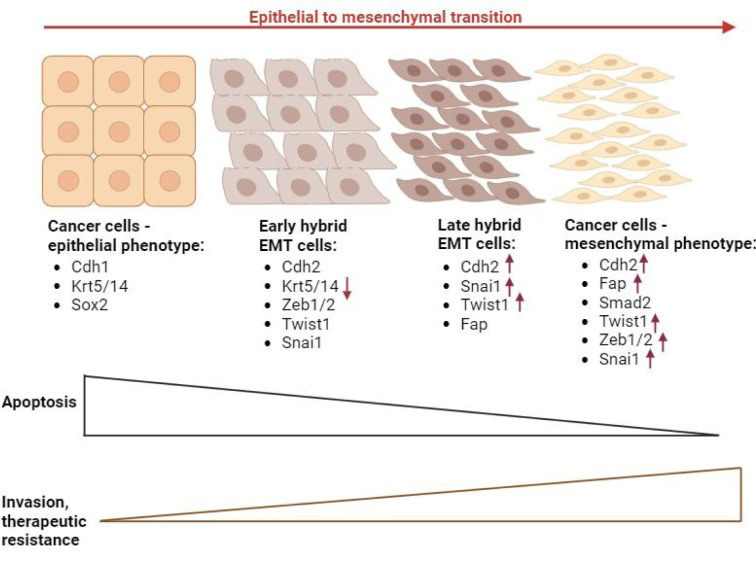
Phenotypic plasticity of CSCs during EMT (Gupta et al., 2019; Tripathi et al., 2023, changed).

CSCs show a high phenotypic plasticity, closely related to EMT phenomenon and stemness (Gupta et al., 2019[46]; Shibue and Weinberg, 2017[118]; Wang et al., 2015[144], Tripathi et al., 2023[133]). EMT is accompanied by the activation of some genes, such as Snai1, Twist, Zeb, and Cdh2 (Table 2(Tab. 2)). Cdh2 encodes N-cadherin, which differs from E-cadherin, a product of Cdh1. The upregulation of N-cadherin, followed by the downregulation of E-cadherin, is observed in EMT (Loh et al., 2019[81]; Tripathi et al., 2023[133]). Figure 4(Fig. 4) (References in Figure 4: Gupta et al., 2019[46]; Tripathi et al., 2023[133]) presents changes in cell morphology, gene expression, and transcription factors involved in CSC phenotypic plasticity during the EMT process. These cells differ in their apoptosis, invasion ability, and therapeutic resistance. 

The term “epithelial-mesenchymal transition” was first used in 1982 by Elizabeth Dexter “Betty” Hay to refer to a change in the phenotype of non-cancerous cells. Later studies on circulating cancer cells provided data that demonstrated that cells can express both genes associated with epithelial and mesenchymal phenotypes (Lachat et al., 2021[67]). It suggests that these cells undergo EMT at multiple levels. Hence, a hypothesis of hybrid EMT was proposed (Gupta et al., 2019[46]). According to this hypothesis, cells may be at different stages of EMT (epithelial/early hybrid/late hybrid/mesenchymal). They differ in apoptosis, invasion, metastatic potential, and therapeutic resistance (Pastushenko et al., 2018[98]; Tripathi et al., 2023[133]). The intermediate hybrid states show the highest plasticity and potential for metastasis. Differences were also found in the metabolic patterns of CSCs, depending on the epithelial/mesenchymal phenotype (Liu et al., 2014[79]; Luo and Wicha, 2015[83]; Warrier et al., 2023[148]). Studies on breast cancer stem cells (BCSCs) have shown that cells displaying a mesenchymal phenotype (CD44+CD24-) have a low proliferative potential and are in a slow-cycling state (quiescence). In contrast, ALDH+ BCSCs have an epithelial phenotype characterized by a high proliferative potential (Liu et al., 2014[79]). A study by Zhao et al. (2017[160]) on pancreatic cancer cells shows that both EMT and stemness are promoted by glycolysis accompanied by low levels of reactive oxygen species (ROS). The implication of this is that cells are resistant to chemotherapy.

### CSC resistance to chemo- and radiotherapy

The phenomenon of EMT allows CSCs to migrate and metastase. In such conditions, resistance to therapy is promoted (Gupta et al., 2019[46]). During experimental induction of EMT in cell lines, increased resistance to the activity of chemotherapeutic agents was observed several times (Gupta et al., 2009[45]; Thiery and Sleeman, 2006[132]). Slug expression promotes cell survival by inhibiting the pro-apoptotic protein PUMA (Wu et al., 2015[152]). The resistance is accompanied by increased activity of the ALDH enzyme, which is involved in cell detoxification (Raha et al., 2014[109]). In studies of esophageal cancer, ALDH1+ cells were found to be more resistant to chemotherapy than ALDH1-cells (Li et al., 2021[75]). The resistance of CSCs to therapy can also result from epigenetic changes (Table 2(Tab. 2)).

CSC resistance to chemotherapy is usually associated with increased expression of MDR1 induced by HIF-1 (Table 2(Tab. 2)). Through HIF-1, cancer cells trigger multiple signaling pathways, DNA repair mechanisms, and autophagy, which determines resistance to chemotherapy and radiotherapy (Olivares-Urbano et al., 2020[96]; Xia et al., 2018[153]). Radioresistance is one of most intriguing and challenging aspects of CSC biology (Hoque et al., 2023[53]. CSCs are able to withstand the deleterious effects of ionizing radiation, rendering them less susceptible to conventional radiotherapeutic strategies (Piotrowski et al., 2022[103]). Radiotherapy, a cornerstone in cancer treatment, mainly eliminates non-CSCs, but the number of CSCs increases (Jin et al., 2020[56]; Martins-Neves et al., 2018[88]; Schulz et al., 2019[114]).

In breast cancer, scientists demonstrated the contribution of the WNT/β-catenin signaling pathway to repopulation and self-renewal of CSCs after radiotherapy (Woodward et al. 2007[151]). Tanaka et al. (2019[129]) found a relationship between increased nuclear accumulation of β-catenin and radioresistance in colon cancer cell lines. Moreover, those cells had a notably elevated proportion of putative CSCs. The radioresistance observed in CSCs from mucoepidermoid carcinoma was activated by the NFκB signaling pathway (Wagner et al. 2016[138]), which regulates inflammation and cell survival. Regarding radioresistance, this pathway also mediates DNA double-strand bond repair and cell-cycle arrest (Tan et al. 2019[128]). The PI3/Akt/mTOR pathway is related to NFκB signaling since it modulates its downstream effectors (Liu et al. 2020[77]). The PI3/Akt/mTOR pathway also facilitates antioxidant mechanisms and induces quiescence CSCs, which is crucial in initiating radiation-induced autophagy. Recent evidence has shown that the concurrent inhibition of both PI3K and mTOR overcomes radioresistance and enhances the effectiveness of treatment (Olivares-Urbano et al., 2020[96]). 

The hypoxic tumor microenvironment (TME) has significant implications for the behavior of CSCs, as it promotes radioresistance. The lack of oxygen can create a protective environment, making CSCs less susceptible to the damaging effects of radiation therapy (Telarovic et al. 2021[130]). In hypoxic niches, where oxygen is minimal, CSCs increase the expression of ROS scavengers. The downregulation of ROS levels triggers the activation of the HIF signaling pathway (Arnold et al. 2020[7]). The activation of HIF can contribute to radioresistance by stimulating cell survival mechanisms and mediating cell cycle, energy metabolism, EMT (by promoting CSC-like cells), autophagy, DNA damage response, epigenetic factors, and cytoprotection from apoptosis (Kabakov and Yakimova, 2021[59]). 

### Immunosuppression

CSCs can induce immunosuppression in the TME, leading to CSC resistance to immunotherapy (Tsuchiya and Shiota, 2021[134]; Zhang et al., 2021[159]). CSCs use multiple mechanisms to trigger tumor immunosuppression. For example, Nanog activation modulates TGF-β expression, followed by induction of immunosuppressive cells, such as regulatory T cells (Tregs) and macrophages with M2 phenotype (Hajizadeh et al., 2019[47]; Zhang et al., 2021[159]). 

Another method to induce immunosuppression involves the reduction of tumor-associated antigens (TAA) or tumor-specific antigens (TSA) through the impairment of antigen processing and presenting machinery. (Tsuchiya and Shiota, 2021[134]). MDSCs (myeloid-derived stem cells) produce and release arginase-1 (Arg-1), which is involved in cytotoxic T cell (Tcyt) dysfunction (Cao et al., 2016[19]). Under L-arginine supplementation, an inhibition of breast cancer growth was observed. The immunosuppressive TME is also characterized by the upregulation of immune checkpoint molecules like PD-L1 (Tsuchiya and Shiota, 2021[134]). The presence of PD-L1 is directly responsible for the anergy of Tcyt. The study of Noman et al. (2014[93]) showed that hypoxia induces PD-L1 expression in tumor cells, MDSCs, dendritic cells, and macrophages. However, PD-L1 expression in CSCs is higher than in other cells, stimulating EMT and increasing metastasis capacity (Rouzbahani et al., 2022[113]). PD-L1 can be delivered as a soluble factor, carried by TEXs (tumor-derived exosomes) (Czystowska-Kuzmicz and Whiteside, 2021[30]; Xie et al., 2022[154]), or CSC-Exos (Yang and Teng, 2023[156]). As a result, M2 polarization is promoted, Tregs are activated, and antigen-presenting cells undergo maturation disorders.

### Epigenetic changes in CSCs 

Epigenetic changes are highly involved in CSC plasticity (Bryl et al., 2022[17]; Khan et al., 2019[62]; Loh et al., 2019[82]; Makowska et al., 2023[85]). These changes do not result from DNA sequence disturbance, but from gene expression regulation via different mechanisms, namely DNA demethylation, chromatin modification, and ncRNA activity. DNA demethylation is crucial for CSC generation, tumor induction, and growth (Warrier et al., 2023[148]). Loss of methylation on the NANOG promoter leads to CSC formation from non-CSCs (Liu et al., 2020[78]). miRNAs belong to ncRNAs and can regulate gene expression posttranscriptionally by interacting with mRNA. miRNAs can have an oncogenic or suppressor function. 

For oncogenic miRNAs, an increase in their expression (up-regulation) is characteristic, while for suppressor miRNAs, a decrease (down-regulation) is observed (Loh et al., 2019[82]). Table 3(Tab. 3) (References in Table 3: Balandeh et al., 2021[11]; Mahinfar et al., 2022[84]; Makowska et al., 2023[85]; Loh et al., 2019[82]; Nejad et al., 2021[91]) summarizes examples of miRNAs regulating various processes of glioblastoma multiforme and breast cancer. For example, in glioblastoma multiforme, the group of oncogenic miRs includes miR21, miR10b, miR93, miR221, miR222, and miR182, while suppressor activity is demonstrated by miR181a, miR181b, miR34a, miR146b, miR124, miR137 or miR128 (Makowska et al., 2023[85]; Piwecka et al., 2015[104]). In breast cancer, the role of oncogenic miRNAs plays miR492, miR135b, miR200c, miR200b, miR141, and miR21. Suppressive effects show miR497, miR16, miR34a, miR455, and miR204-5p (Loh et al., 2019[82]). 

The increase in expression of selected miRNAs observed in CSCs under the influence of hypoxia may lead to the resistance of cancer cells to treatment. Studies on esophageal squamous cell carcinoma (Peng et al., 2020[102]) have shown a close correlation between miR21 levels and PTEN expression. High levels of miR21 lead to the inhibition of PTEN activity, resulting in radiation resistance through increased proliferation and reduced apoptosis. In non-small cell lung cancer, miR-410 overexpression promotes EMT, radioresistance, and enhanced DNA damage repair (Yuan et al., 2020[157]).

The activity of miRNAs can be regulated via circular RNAs (circRNAs) and long non-coding RNAs (lncRNAs) (Bryl et al., 2022[17]). circRNAs can bind with miRNAs and thus prevent its interaction with the target, acting as a sponge (Ahmed et al., 2022[1]; Bryl et al., 2022[17]). Similar to miRNAs, circRNAs can act as suppressors or oncogenic factors. For example, circRGPD6 exhibits a suppressor effect in breast cancer in CSCs, while hsa_circ_002178 shows a promoter effect. lncRNAs can also interact with miRNAs. The concentration of the lncRNA H19 promoter is much higher in the cytoplasm than in the nucleus, due to its interactions with the miRNA let-7 (Peng et al., 2017[101]) which results in the increased expression of HIF-1α. In addition to H19, the lncRNA group also includes HOTAIR, Linc-ROR, and Linc00152. Like miRNAs, lncRNAs affect many signal pathways, including TGF-β, Wnt, PI3K/Akt, and HIF-1 (Heery et al., 2017[51]). 

ncRNAs can promote CSC dormancy (Bryl et al., 2022[17]). The increase in ncRNA expression accompanies high expression of HIF-1 (Francescangeli et al., 2023[40]). Silenced cancer cells have a reduced metabolism, do not divide, but retain their ability to proliferate and eliminate DNA damage (Carcereri de Prati et al., 2017[21]; Cho et al., 2019[27]). This condition is usually reversible and is known as quiescence. For example, in lung cancer, lncRNA NR2F1-AS1 regulates NR2F1, promoting quiescence (Liu et al., 2021[80]). ncRNAs derived from exosomes of microenvironmental cells may also be responsible for inducing the dormancy of CSCs. Studies using the MDA-MB-231 breast cancer cell line demonstrated an interaction between CSCs and mesenchymal stem cells via exosomal miR 222-223 and induced dormancy (Bliss et al., 2016[14]). CSC-Exos are also essential for communication with other cells in TME, including non-CSC cells (Yang and Teng, 2023[156]). CSC-Exos transport stemness-related factors, like NANOG or ncRNAs. For example, miR-19b-3p can be carried into cancer cells, which leads to EMT induction and facilitates metastasis (Wang et al., 2019[142]). 

## CSCs as a Target of Therapy – Still a Big Challenge?

Recent years have shown that drugs such as salinomycin may contribute to the elimination of CSCs (Huczynski et al., 2016[54]). Salinomycin is an ionophore antibiotic isolated from the bacterial strain Streptomyces albus. The effects of salinomycin include interfering with the Na^+^/K^+^ ion balance, inhibiting the Wnt pathway, increasing caspase activity, activating the MAPKp38 pathway, and inhibiting NF-κB (Huczynski et al., 2016[54]). The main effect is the induction of cell apoptosis. Studies using the CD44^high^/CD24^low^ breast cancer cell line revealed that the antibiotic is almost 100 times more effective in eliminating CSCs compared to drugs such as paclitaxel (Gupta et al., 2009[45]). Due to its molecule size, salinomycin is not subject to pumping out of the cell via the MDR1 protein, and actually inhibits this protein. Hence, salinomycin can induce apoptosis in those cells that have acquired resistance through high expression of MDR1 and anti-apoptotic proteins such as bcl-2 (Dewangan et al., 2017[33]). Regarding the crucial role of CSC interactions in the TME, CSCs-derived Exos seem to be a good target for innovative therapies (Yang and Teng, 2023[156]). 

Some surface markers of CSCs may be used as a target for anti-CSC therapy (Eid et al., 2023[37]). Current attention is on anti-EpCAM antibodies (Catumaxomab) (Kubo et al., 2018[66]), or regulation of CD133 levels by the beta-2 isoform of PLC (PLC-β2) (Brugnoli et al., 2019[16]).

Surface antigens of cancer cells have become a tool of innovative CAR-T cell therapy. The groundwork for CAR-T (chimeric antigen receptor) therapy began in 1989 when the “two-chain” CAR receptor antibody TCRaV H + TCRbV L was first obtained *in vitro*, followed in 1993 by the first cancer-specific CARs demonstrating *in vitro* cytotoxicity (Eshhar, 2014[38]; Styczynski, 2020[123]). These findings are the basis of the development and subsequent use of the first CAR-T cell drugs targeting CD19, present on the surface of acute lymphoblastic leukemia and lymphoma cells. Currently, markers present on the surface of solid tumor cells, like EGFR, Her-2, Muc16, CEA, or TAA, are tested in preclinical and clinical trials (Marofi et al., 2021[87]; Masoumi et al., 2021[89]; Veschi et al., 2023[136]). 

The problem in developing an optimal CAR-T construct that acts selectively on CSCs is that some CSC markers are also present on the surface of normal stem cells. However, the results of phase I clinical trial NCT02541370 (Wang et al., 2018[145]) demonstrated the positive effect of CD-133^+^-CAR-T in patients with hepatocellular carcinoma, and metastasis did not develop. In thyroid cancer studies, the phosphorylation in CD133^+^CSCs promotes their self-renewal and immune escape via induction of PD-L1 expression (Wang et al., 2020[146]). High expression of PD-L1 interacting with PD-1 on Tcyt can induce anergy of both CAR-T cells and non-genetic modified T cells (Johnson et al., 2022[57]). Targeting only immune checkpoints, including PD-L1, is insufficient to achieve the full efficacy of anti-tumor therapy. Combination therapy using immune checkpoint inhibitors with chemotherapy (Grodzka et al., 2023[43]) or with CAR-T cells (Dianat-Moghadam et al., 2022[34]) is recommended.

The immunosuppressive microenvironment and tumor cells' high plasticity can be significant obstacles to CAR-T cell effectiveness (Table 4(Tab. 4); References in Table 4: Cheever et al., 2022[26]; Masoumi et al., 2021[89]) (Masoumi et al., 2021[89]). Increasing TAA expression and using bispecific CAR-T can be helpful (Catamero et al., 2022[22]). Many clinical trials are being conducted with this aim, among them is a phase I/II clinical trial (NCT04077866) with an intratumorally administered B7-H3-CAR-T construct in patients with glioblastoma multiforme. A phase I clinical trial, NCT04227275, with CAR-T-PSMA-TGFβRDN cells, is dedicated to prostate cancer patients (Kankeu Fonkoua et al., 2022[61]). These CAR-T cells have information about the prostate-specific membrane protein antigen (PSMA) and TGFβRDN. As a result, the block of TGFβ immunosuppressive effects is observed. This action may contribute to “bypassing” the immunosuppressive effects of the microenvironment (Cheever et al., 2022[26]).

Combining CAR-T cells with epigenetic reprogramming, epi-immunotherapy is now considered a promising research direction (Veschi et al., 2023[136]). Such compounds as DNMT inhibitors, BET, HDAC, EZH, and SIRT-1 agonists, among others, have received attention. DNMTi modulates macrophage activity and promotes Tcyt activity, and BET reduces PD-L1 expression. Acute myeloid leukemia studies suggest that DNMTi may also increase TAA expression on the surface of tumor cells, which may provide greater CAR-T cell efficacy in target recognition (Veschi et al., 2023[136]). A similar effect was described by Kailayangiri and colleagues (2019[60]) after using CAR-T cells specific for GD2, a marker of Ewing's sarcoma tumor cells. Regarding CAR-T cell effectiveness, epi-immunotherapy opens possibilities for epigenetic reprogramming with modifiers acting via miRNAs at the DNA level (Alvanou et al., 2023[4]; Li et al., 2016[74]). In addition to miRNA-based drugs targeted to cancer cells (Bryl et al., 2022[17]), ncRNAs, like miR-28, can be used to protect T cells from anergy by silencing PD-1 and regulating cytokine secretion. miRNA138 can silence PD-1, as well as CTLA-4 (Akbari et al., 2021[2]). Another example is miRNA155, whose expression in CAR-T cells increases the cytolytic activity of CD19-CAR-T cells (Zhang et al., 2022[158]). 

## Conclusions

Much research over the last few decades has led to the breakthrough discovery of CSCs, their biology, and their role in tumor initiation, development, metastasis, as well as cancer resistance to treatment. However, the origin of cancer stem-like cells is still unclear, and the CSC model is still in debate. The lack of consensus on reliable CSC markers and definition complicates translating the study results into clinical applications. For this reason, further research on CSC and cancer cell heterogeneity should be a priority. The most promising therapeutic approach is the development of combination therapies in which a cancer stem-like cell-targeted strategy could be used with chemotherapy, radiation therapy, and/or immunotherapy. There are high hopes for strategies based on ncRNAs or genetically modified T cells. However, researchers and clinicians may face many limitations and challenges regarding the complex biology and dynamic changes of cancer cells and within the tumor microenvironment.

## Declaration

### Conflict of interest

The authors declare that they have no conflict of interest.

### Acknowledgments

The authors would like to thank Joshua E. Donovan from the University of Pittsburgh United States for linguistic proofreading of the article.

## Figures and Tables

**Table 1:**
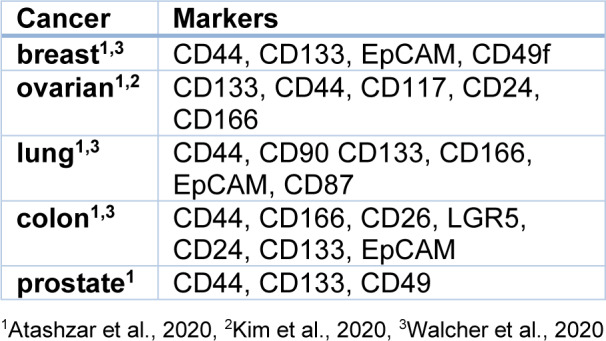
Surface CSC markers

**Table 2:**
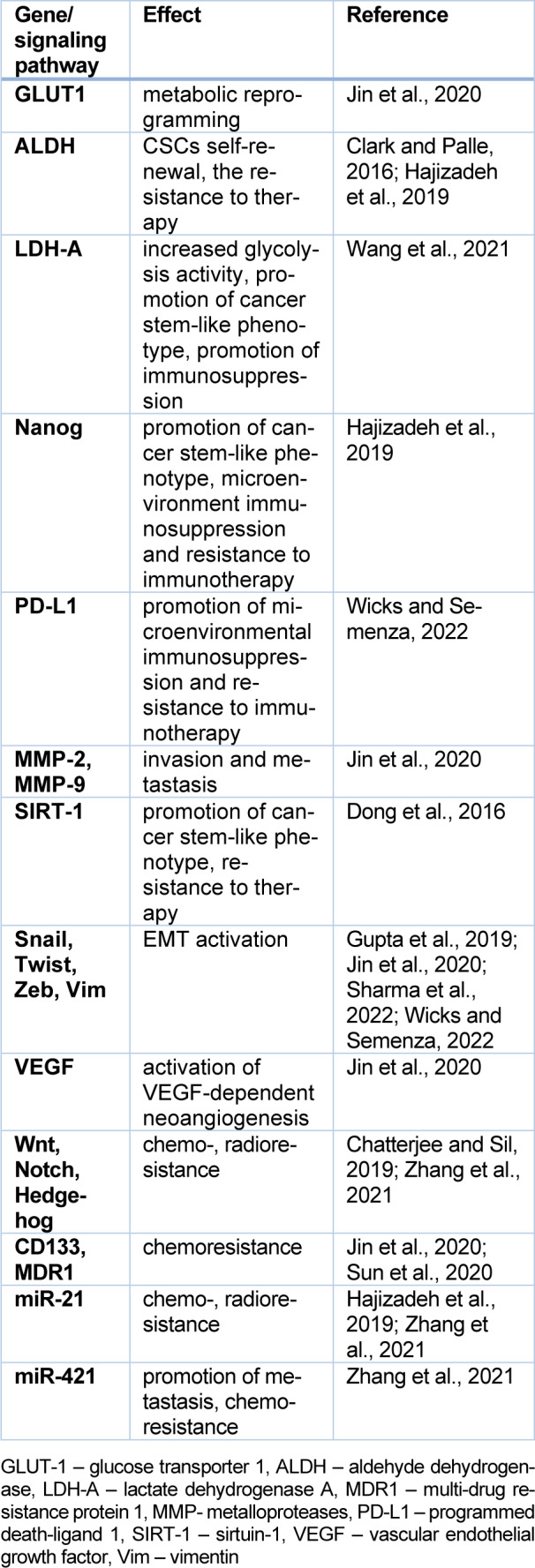
Hypoxia-induced gene expression in CSCs

**Table 3:**
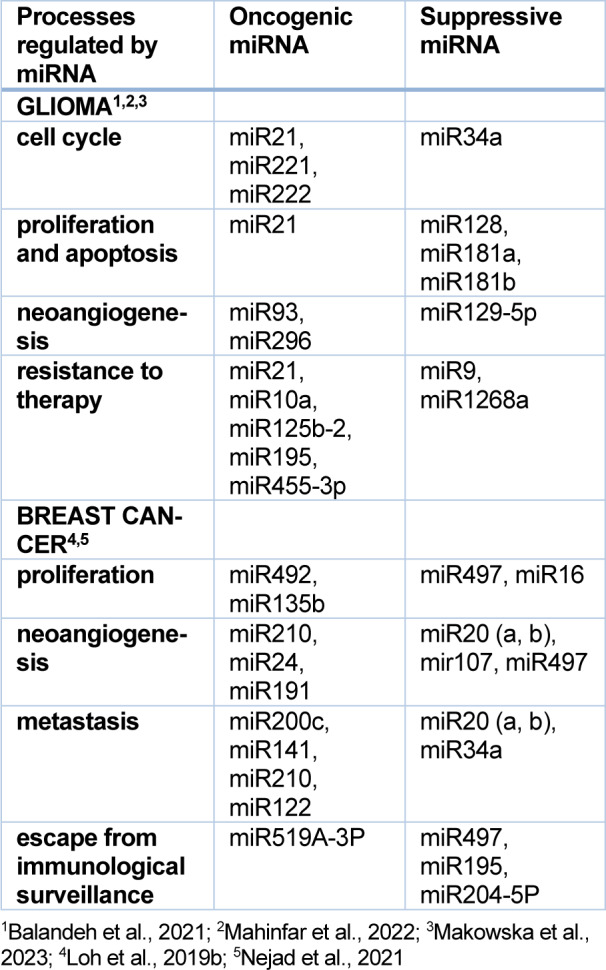
miRNA associated with glioma and breast cancer

**Table 4:**
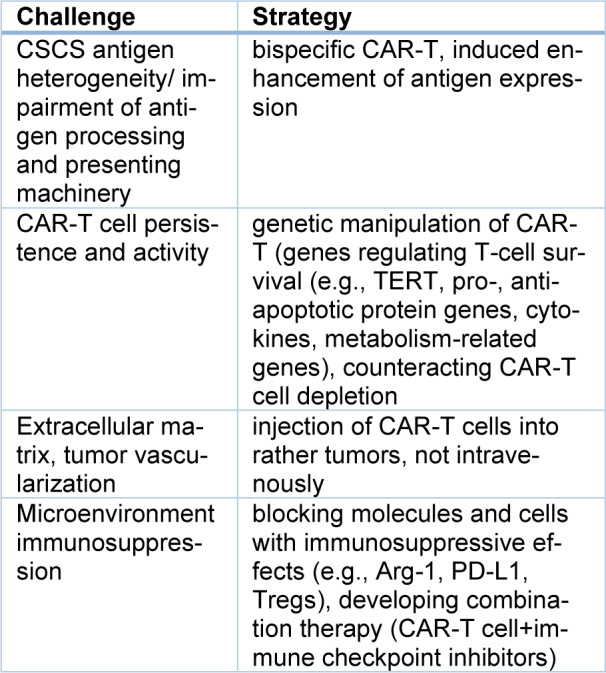
Challenges for CSC-targeted CAR-T cell therapy (Cheever et al., 2022; Masoumi et al., 2021, changed).

**Figure 1:**
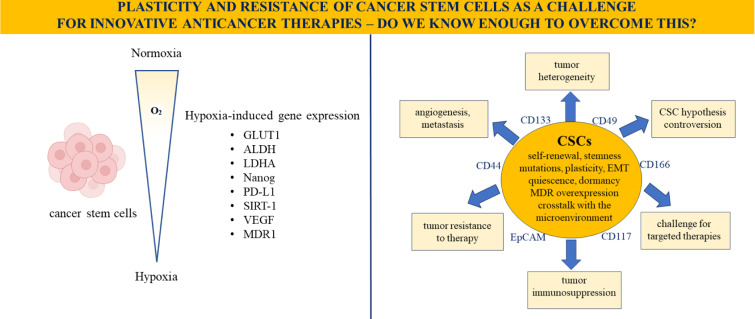
Graphical abstract
